# ADAPTING AUTOETHNOGRAPHY TO EXPLORE CULTURAL AND GENERATIONAL DIFFERENCES ON AGING

**DOI:** 10.1093/geroni/igad104.2173

**Published:** 2023-12-21

**Authors:** Mohammad Hossain, Natalie Pope

**Affiliations:** University of Kentucky, Lexington, Kentucky, United States; University of Kentucky, Lexington, Kentucky, United States

## Abstract

This presentation uses adapted autoethnography to explore cultural and generational differences on aging identity and care for older adults. Our presentation is based on an oral history of Virginia Bell, a gerontological social worker and dementia expert who developed an innovative model of dementia care known as the Best FriendsTM approach (Bell & Troxel, 2012; 2016). Through several in-depth interviews, this oral history project created space for conversation across generations – discussions between two cisgender white women, both social workers and gerontologists. At age 98, Bell reflected on people and experiences that shaped her perspective on caring for people with dementia from a person-centered, relational approach. The interviewer, in her mid-40s, assumed a co-interpreter role with Bell, documenting her childhood and formative years, as well as what informed her approach to caring for people with dementia. In this presentation, we bring a third voice into the conversation. During data analysis, a first-year graduate student was invited to help code and analyze Bell’s interview transcripts. As a novice gerontologist and social worker from Bangladesh, this cisgender male in his 40s shares his reactions to Bell’s oral history interviews. Specifically, this autoethnography-inspired presentation examines cultural and generational differences on aging identity and cultural norms on care for older adults. We compare and contrast the personal/ professional experiences of Bell and this Bangladeshi student while situating their reflections in extant literature. This project highlights the utility of autoethnography to systematically analyze individuals’ experiences with aging embedded in a larger social and cultural context.

